# Is Intraoperative Bleeding Control Useful After Tourniquet Release in Arthroscopic Anterior Cruciate Ligament Reconstruction?

**DOI:** 10.7759/cureus.44253

**Published:** 2023-08-28

**Authors:** Oğuzhan Çimen, Ibrahim Azboy, Bertan Cengiz, Mehmet Çavuş, Sinan Karaoglu

**Affiliations:** 1 Orthopaedics and Traumatology, Medistanbul Hospital, Istanbul, TUR; 2 Orthopaedics, Medipol University, Istanbul, TUR; 3 Orthopaedics and Traumatology, Acibadem Kayseri Hospital, Kayseri, TUR

**Keywords:** coagulation, bleeding control, tourniquet, hemarthrosis, anterior cruciate ligament

## Abstract

Background

Arthroscopic anterior cruciate ligament (ACL) reconstruction is a common orthopedic surgery, and rehabilitation is very important to achieve successful postoperative results. Postoperative hemarthrosis causes pain and limitation of movement, which prolongs the rehabilitation period. For these reasons, various strategies are used to reduce hemarthrosis in patients undergoing ACL reconstruction. This study aimed to evaluate the effect of bleeding control after releasing the tourniquet in ACL reconstruction surgery on the amount of hemarthrosis and pain in the postoperative period.

Methodology

A total of 60 patients who underwent arthroscopic single-bundle ACL reconstruction were enrolled in this prospective randomized control study. Bleeding control with the radiofrequency (RF) probe after releasing the tourniquet was done at the end of the arthroscopic ACL reconstruction in 30 patients (coagulation group) while bleeding control was not done for the other 30 patients (control group). Both groups were compared in terms of the degree of hemarthrosis using the Coupens and Yates classification in the early postoperative period and the degree of pain using the Visual Analog Scale (VAS) score and postoperative complications.

Results

In both groups, isolated ACL reconstruction was performed in 10 patients, additional partial meniscectomy in three patients, and additional arthroscopic meniscus repair in 17 patients. There was no statistically significant difference between the coagulation and control groups in terms of VAS (p > 0.05) and the degree of hemarthrosis (p > 0.05). Although the duration of tourniquet application was similar in both groups (p = 0.78), the duration of anesthesia was significantly longer in the coagulation group (p = 0.001). There was no significant difference between the groups in terms of postoperative complications.

Conclusions

Bleeding control with the RF probe after tourniquet release does not yield superior outcomes. More research with larger populations is needed to confirm these findings.

## Introduction

Arthroscopic anterior cruciate ligament (ACL) reconstruction is a common orthopedic surgery, and rehabilitation is very important to achieve successful postoperative results [1]. Postoperative hemarthrosis causes pain and limitation of movement, which prolongs the rehabilitation period [2-5]. In addition, hemarthrosis can cause undesirable complications such as infections, possible toxic effects on cartilage, synovitis, and arthrofibrosis, which can also lead to an increase in the length of stay and an increase in healthcare costs [2,3]. In the case of severe hemarthrosis, knee arthrocentesis may be required to alleviate pain and restore knee range of motion. This is an invasive and painful procedure that is poorly tolerated by patients and may also result in an increased risk of septic arthritis [6].

Hemarthrosis develops in 3-10% of patients undergoing ACL reconstruction [2], and various strategies have been attempted to prevent this complication. Tourniquets, drains, and compressive bandages have been used for a long time [6,7]. In recent years, the use of intravenous or intra-articular tranexamic acid (TXA) has become widespread to reduce hemarthrosis after arthroscopic ACL reconstruction [8-10]. To our knowledge, there is no study in the literature reporting bleeding control after releasing the tourniquet in ACL reconstruction and evaluating the amount of postoperative pain and hemarthrosis.

We conducted a prospective randomized study with the hypothesis that after performing arthroscopic ACL reconstruction, releasing the tourniquet and controlling bleeding will decrease the amount of hemarthrosis in the early postoperative period.

## Materials and methods

A prospective randomized controlled study was planned after obtaining ethical approval from the Istanbul Medipol University Non-interventive Clinical Trials Ethics Committee (approval number: E-10840098-772.02-2971). A total of 60 patients who underwent arthroscopic single-bundle ACL reconstruction were enrolled in this study. Patients were allocated randomly to one of two groups using an envelope with an opaque seal. Bleeding control after releasing the tourniquet was performed at the end of arthroscopic ACL reconstruction in 30 patients (coagulation group). The artery between the ACL and the posterior cruciate ligament, namely, the middle geniculate artery, was controlled, and if bleeding was observed, it was coagulated with a radiofrequency (RF) probe. Bleeding vessels in Hoffa were coagulated using the RF probe. Bleeding control was not applied to the other 30 patients (control group). Exclusion criteria were bleeding and coagulation disorders, preoperative use of anticoagulants, preoperative range of motion <100°, multi-ligament injury, revision ACL reconstruction, patients who underwent microfracture, and patients with an injury duration of less than three weeks (because there would already be a hematoma in the knee).

Patient age, gender, tourniquet time, anesthesia time, length of stay, and additional procedures such as meniscectomy and meniscus repair were recorded. All ACL reconstructions were performed using hamstring tendon autografts with the same single-incision technique under tourniquet control. Tourniquet pressure was adjusted to be 150 mmHg higher than systolic blood pressure. Intra-articular suction drain was not used in any case. Postoperative Jones compression dressing was applied to patients in the operating room, and ice was routinely applied to patients for 20 minutes every two hours for two days.

All patients received 1,000 mg paracetamol (maximum four times a day) and 100 mg tramadol HCl (twice a day) in the postoperative period, and the drug doses used were recorded. No patient was given non-steroidal anti-inflammatory drugs during the postoperative period.

The MOON ACL rehabilitation protocol was applied to all patients [11]. ACL braces were not used. Patients were mobilized in the early postoperative period, as tolerated. Deep vein thrombosis prophylaxis was initiated with aspirin 100 mg 12 hours postoperatively and was continued once a day for one month. All patients (except one) were discharged on the second postoperative day. All patients were discharged on the same type of analgesic. Complications were recorded. All arthroscopic ACL reconstruction surgeries were performed by a single orthopedic surgeon (SK).

Evaluation of clinical outcomes

The primary outcome measures were to compare both groups in the early postoperative period for the degree of hemarthrosis according to the Coupens and Yates classification [12] (Table 1) and the degree of pain using the Visual Analog Scale (VAS). Evaluations were made on postoperative days one, two, seven, 14, and 45. The thigh circumference was also measured for the evaluation of hemarthrosis. The thigh circumference was measured 2 cm proximal to the superior pole of the patella when the knee was in full extension [13]. Tight circumference was measured preoperatively and on days one, two, and seven postoperatively. If hemarthrosis caused knee flexion limitation on the first postoperative day, needle aspiration was performed [14]. All patients in both groups were followed for 45 days.

**Table 1:** Clinical grading of hemarthrosis (Coupens and Yates classification).

Grade	Description
0	No detectable fluid
1	Fluid present with a fluid wave
2	Palpable fluid in the suprapatellar space
3	Ballotable patella
4	Tense hemarthrosis

Statistical analysis

Power analysis was performed using a general power analysis program (G*Power 3.1.9.4, Kiel, Germany). Power calculation was used to determine the minimum sample size assuming an alpha of 0.05 at 80% power based on an effect size of 0.52. A sample size of 29 patients per group was calculated [15].

In the descriptive statistics of the data, mean, standard deviation (SD), lowest, highest, frequency, and ratio values were used. The distribution of variables was determined using the Kolmogorov-Smirnov test. Analysis of variance (Tukey test) was used in the analysis of quantitative independent data, and t-test, Kruskal-Wallis test, and Mann-Whitney U test were used for the evaluation of independent samples. Paired-sample t-test and Wilcoxon test were used for the analysis of dependent quantitative data. The chi-square test was used in the analysis of qualitative independent data, and Fisher test was used when the chi-square test conditions were not met. SPSS version 28.0 (IBM Corp., Armonk, NY, USA) program was used for data analysis.

## Results

There were 27 males in the coagulation group and 25 males in the control group. The mean age of the patients in the coagulation group was 28.3 years (range = 18-50 years) and 32.3 years (range = 18-48 years) in the control group. The mean patient age, operative time, length of stay, and sex distribution were similar between the groups. In both groups, isolated ACL reconstruction was performed in 10 patients, additional partial meniscectomy in three patients, and additional arthroscopic meniscus repair in 17 patients (Table 2). There was no statistically significant difference in pain scores (p > 0.05) and degree of hemarthrosis (p > 0.05) between both groups (Table 3). In addition, there was no difference between the groups in terms of thigh circumference measurement and postoperative analgesic requirements (p > 0.05). The duration of tourniquet application was similar in both groups (p = 0.78), but the duration of anesthesia was significantly longer in the coagulation group (p = 0.001) (Table 4).

**Table 2:** Characteristics of included study patients at the time of admission. ACL: anterior cruciate ligament; ROM: range of motion

Variables	Coagulation group	Control group	P-value
N (male/female)	27/3	25/5	0.056
Age (year)	28.3 ± 8.7	32.3 ± 7.2	0.448
Injury type			
ACL rupture only (n)	10	10	1.000
ACL rupture and meniscus injury (n)	20	20	
Preop ROM (degree)	134.8 ± 1.25	135.0 ± 0.0	0.317
Length of stay in the hospital (day)			
1	1	0	1.000
2	29	30	
Surgery			
Isolated ACL reconstruction (n)	10	10	1.000
Partial meniscectomy (n)	3	3	
Meniscus repair (n)	17	17	

**Table 3:** Comparison of groups according to the Coupens and Yates Classification and knee arthrocentesis procedure. PO: postoperative

Coupens and Yates classification	Coagulation group, N (%)	Control group, N (%)	P-value
PO day 1			
1	22 (73)	26 (87)	0.197
2	6 (20)	4 (13)	0.488
3	2 (7)	0 (0)	0.492
PO day 2			
1	25 (84)	27 (90)	0.448
2	4 (13)	3 (10)	0.688
3	1 (3)	0 (0)	1.000
PO day 7			
0	3 (10)	2 (7)	0.640
1	23 (77)	23 (77)	
2	4 (13)	5 (16)	
PO day 14			
0	16 (53)	18 (60)	0.602
1	14 (47)	12 (40)	
PO day 45			
0	26 (87)	26 (87)	1.000
1	4 (13)	4 (13)	
Knee arthrocentesis procedure (n)			
0	28 (93)	26 (87)	0.389
1	2 (7)	3 (10)	
2	0 (0)	1 (3)	

**Table 4:** Results of the patients in the coagulation and control groups. PO: postoperative

Variables	Coagulation group, mean ± SD	Control group, mean ± SD	P-value
Tourniquet time (minute)	38.9 ± 16.4	37.1 ± 12.3	0.778
Anesthesia time (minute)	79.5 ± 20.8	63.4 ± 16.0	0.001
VAS			
PO day 1	3.6 ± 1.3	4.0 ± 1.0	0.110
PO day 2	3.1 ± 1.3	3.3 ± 1.2	0.457
PO day 7	2.4 ± 1.2	1.9 ± 1.1	0.101
PO day 14	1.3 ± 1.1	1.0 ± 1.0	0.239
PO day 45	0.2 ± 0.5	0.2 ± 0.5	0.750
Range of motion			
Preoperative	134.8 ± 0.9	135.0 ± 0.0	0.317
PO day 1	92.4 ± 2.7	92.3 ± 4.2	0.987
PO day 2	102.1 ± 5.6	100.9 ± 5.4	0.661
PO day 7	116.2 ± 5.3	115.0 ±.7.1	0.508
PO day 14	125.9 ± 6.1	125.2 ± 7.9	0.803
PO day 45	135.0 ± 0.0	135.0 ± 0.0	1.000
Thigh circumference (cm)			
PO day 1	44.5 ± 4.2	44.9 ± 5.0	0.773
PO day 2	47.4 ± 6.6	45.0 ± 5.0	0.894
PO day 7	43.5 ± 4.4	43.9 ± 4.6	0.888
Tramadol HCl dose used (mg)			
Day 1	253.3 ± 73.0	276.7 ± 67.9	0.139
Day 2	160 ± 107.0	180.0 ± 99.7	0.484
Paracetamol total dose (mg)			
Day 1	2930 ± 870	3000 ± 830	0.664
Day 2	1630 ± 560	1930 ± 870	0.128

In the control group, synovitis developed in one patient in the postoperative period for which arthroscopic debridement was performed. In the early postoperative period, 10° of extension restriction developed in one patient in the coagulation group and in two patients in the control group, and improvement was observed in all of them in 45 days.

## Discussion

This study demonstrates that bleeding control after releasing a tourniquet in patients undergoing arthroscopic ACL reconstruction does not reduce the amount of hemarthrosis and the severity of pain in the early postoperative period.

Various modalities have been used for the prevention of hemarthrosis after arthroscopic ACL reconstruction, such as tourniquets, drains, and compressive bandages, but none of the modalities has been established as a gold standard. Nakayama and Yoshiya [4] in a study of 51 patients comparing the use of a tourniquet and no tourniquet found that intra-articular bleeding was significantly higher in the tourniquet group during the early postoperative period. In a systematic review, Clifton et al. [5] reported that the use of drains in ACL reconstruction did not reduce postoperative bleeding. In a study of 60 patients comparing the use of compression dressing with no compression dressing, Coupens and Yates [12] found compressive bandages have no effect on reducing postoperative hemarthrosis. While there are studies reporting that the use of TXA reduces the amount of hemarthrosis in the early postoperative period [10,16,17], studies have also reported that it has no effect on the amount of hemarthrosis [18,19].

The cause of hemarthrosis after arthroscopic ACL surgery is cartilage and soft tissue procedures, bone tunnels, and notchplasty area; however, bleeding is mainly due to cancellous bone bleeding [20]. Pape et al. [21] compared blood loss after ACL reconstruction with and without notchplasty. They divided 58 consecutive patients undergoing primary ACL reconstruction into two groups: 21 received notchplasty, and 37 did not. They reported 30% more blood loss in the notchplasty group. Bahl et al. [2] in a prospective study with 100 patients compared double-band and single-band ACL reconstruction with regards to the pain and hemarthrosis postoperatively. They reported that double-band ACL reconstruction developed more hemarthrosis than single-band ACL reconstruction. However, to our knowledge, the effect of coagulation on bleeding after tourniquet release has not been investigated. We performed this study with the hypothesis that releasing the tourniquet after ACL reconstruction and coagulating the bleeding vessels in the notch region will decrease the degree of hemarthrosis and severity of pain in the postoperative period. However, we observed that this coagulation procedure did not affect the amount of hemarthrosis and VAS score in the postoperative period, and even prolonged the anesthesia time of the patient during the operation. In light of the data of our study, we believe that the cause of hemarthrosis developing after arthroscopic ACL reconstruction surgery is not bleeding in the notch or Hoffa. We consider that the main cause of hemarthrosis is cancellous bone bleeding from bone tunnels. Therefore, optimal tunnel width and tight fit of the graft in the tunnel may prevent postoperative hemarthrosis.

Limitations

There are some limitations of our study. First the Coupens and Yates classification used to evaluate hemarthrosis is not a quantitative criteria. The sample size was relatively small. Another limitation is the lack of evaluation of the relationship between tunnel diameters and bleeding.

## Conclusions

Bleeding control using the RF probe after tourniquet release did not yield superior outcomes in this study. Further research with larger populations is needed to confirm the findings of this study.
